# Predictive Accuracy of First-Trimester Biochemical and Clinical Markers in the Early Identification of Gestational Diabetes Mellitus: A Prospective Observational Study

**DOI:** 10.7759/cureus.93894

**Published:** 2025-10-05

**Authors:** Prithika B, Revathy T.G, Jahnavi Chandrasekar

**Affiliations:** 1 Obstetrics and Gynaecology, Sree Balaji Medical College and Hospital, Chennai, IND

**Keywords:** crp levels, first trimester screening, gdm- gestational diabetes mellitus, predictive marker, south asian women

## Abstract

Background

Gestational diabetes mellitus (GDM) is a common metabolic complication of pregnancy associated with significant maternal and fetal morbidity. Early identification of women at risk is essential to improve outcomes. This study evaluated the predictive accuracy of first-trimester biochemical and clinical markers for the early detection of GDM in a South Indian population.

Methods

A prospective observational study was conducted at a tertiary care hospital in South India over eight months. A total of 118 antenatal women between 11 and 14 weeks of gestation were enrolled. All participants underwent assessment of serum C-reactive protein (CRP), uric acid, and postprandial blood sugar (PPBS) levels, along with clinical evaluation of body mass index (BMI), history of polycystic ovary syndrome (PCOS), and family history of diabetes mellitus. Between 24 and 28 weeks, all women underwent a 75-gram oral glucose challenge test as per the Diabetes in Pregnancy Study Group India (DIPSI) criteria. GDM was diagnosed if the two-hour plasma glucose was ≥140 mg/dL. Statistical analysis included receiver operating characteristic (ROC) curve analysis, logistic regression, and correlation studies.

Results

The incidence of GDM was 27.9% (31/118). Mean CRP, uric acid, and PPBS levels in the first trimester were significantly higher in the GDM group (CRP: 12.96 ± 5.38 mg/L vs. 5.51 ± 3.12 mg/L; uric acid: 3.31 ± 0.52 mg/dL vs. 3.02 ± 0.28 mg/dL; PPBS: 133.06 ± 13.15 mg/dL vs. 92.56 ± 5.57 mg/dL; all p<0.05). ROC analysis showed high predictive accuracy for CRP (area under the curve (AUC) = 0.94, cut-off >8.5 mg/L) and PPBS (AUC = 0.85, cut-off >110 mg/dL). Logistic regression identified CRP (OR: 6.21), PPBS (OR: 4.76), history of PCOS (OR: 3.66), and family history of diabetes (OR: 3.45) as independent predictors of GDM. Among the evaluated biomarkers, CRP (>8.5 mg/L) showed the highest diagnostic accuracy with a sensitivity of 83.9% and specificity of 87.4%, followed by PPBS (>130 mg/dL) with a sensitivity of 80.6% and specificity of 85.1%, while uric acid (3.3 mg/dL) demonstrated comparatively lower diagnostic performance. A combined model of CRP, PPBS, and clinical risk factors achieved excellent predictive value (R² = 0.79).

Conclusion

First-trimester CRP and PPBS, alongside PCOS and family history, were associated with DIPSI-defined GDM in a single-centre cohort; uric acid was not independently predictive. These preliminary results require multicentre external validation and clinical-utility assessment before any screening or management recommendations can be made. Larger, multicentric studies are warranted to confirm these findings and to support the integration of multifactorial risk-based screening into routine antenatal care.

## Introduction

Gestational diabetes mellitus (GDM) is defined as any degree of glucose intolerance with onset or first recognition during pregnancy. It continues to be one of the most common medical disorders complicating pregnancy and is associated with a broad spectrum of adverse maternal and neonatal outcomes [1]. The presence of GDM not only increases the risk of perinatal complications such as macrosomia, neonatal hypoglycemia, and birth trauma but also predisposes both mother and child to long-term metabolic disorders, including type 2 diabetes mellitus and cardiovascular disease. With the rising global burden of noncommunicable diseases, GDM has emerged as a significant contributor, particularly in low- and middle-income countries, including India, where lifestyle transitions and rapid urbanization have intensified the challenge.

According to the American Diabetes Association (ADA), timely detection of GDM is crucial for ensuring optimal pregnancy outcomes. Conventionally, screening is recommended during the second trimester, typically between 24 and 28 weeks of gestation [1]. However, this timing coincides with a period when crucial fetal organogenesis is already complete. As a result, the opportunity to initiate early metabolic control and thereby minimize fetal exposure to maternal hyperglycemia is lost. Recent evidence suggests that metabolic changes contributing to GDM often begin much earlier in gestation, even during the first trimester. Detecting and intervening in this early window may provide an opportunity to prevent complications and mitigate the heightened lifetime risk of type 2 diabetes mellitus in mothers and their offspring [2].

To harmonize the diagnosis of GDM across populations, the International Association of Diabetes and Pregnancy Study Groups (IADPSG) proposed consensus-based diagnostic thresholds that have since been endorsed by both the World Health Organization (WHO) and the ADA [2]. These criteria have highlighted the widespread burden of GDM and facilitated comparability across studies. Nevertheless, the prevalence of GDM varies considerably depending on the diagnostic criteria and population under study, with global estimates ranging between 9% and 25% [3].

Ethnic and regional differences play a crucial role in the epidemiology of GDM. Findings from multicenter IADPSG studies reveal significant variation in prevalence across ethnic groups, with Asian populations-and particularly South Asians-demonstrating an increased predisposition [3]. Data from India support this observation. Urban and semi-urban populations in the country have shown particularly high GDM rates, likely reflecting a combination of genetic predisposition, rising obesity, physical inactivity, and dietary transitions. For instance, a South Indian study by Nallaperumal et al. reported that nearly 20% of pregnant women screened positive for GDM using IADPSG criteria [4]. Alarmingly, the International Diabetes Federation estimates that more than five million pregnancies in India are affected by GDM annually, making the condition a critical public health issue [5].

The pathophysiology of GDM is complex, involving an interplay of insulin resistance and inadequate pancreatic beta-cell compensation. During pregnancy, insulin resistance physiologically rises under the influence of placental hormones such as human placental lactogen, progesterone, estrogen, and cortisol. In women with GDM, this natural insulin resistance becomes exaggerated, while beta-cell response remains inadequate, leading to hyperglycemia [6]. Recently, attention has shifted toward early pathophysiological changes preceding clinical hyperglycemia. Studies suggest that endothelial dysfunction, chronic inflammation, and lipid metabolism abnormalities may already be evident in the first trimester. Koukkou et al. demonstrated altered lipid and lipoprotein profiles among women who subsequently developed GDM, indicating early subclinical disturbances [6].

Among potential biomarkers, C-reactive protein (CRP), an established marker of low-grade systemic inflammation, has been widely investigated. Savvidou et al. reported significantly elevated CRP concentrations in women who later developed GDM, suggesting a potential mechanistic link between chronic inflammation and insulin resistance [7]. CRP may impair insulin signaling and promote systemic insulin resistance, contributing to glucose dysregulation. Importantly, CRP testing is cost-effective and stable, making it attractive for large-scale antenatal screening. Qiu et al. provided prospective evidence that elevated maternal CRP levels in the first trimester independently predict GDM risk even after adjusting for age and BMI [8]. Similarly, Wolf et al. confirmed the predictive value of first-trimester CRP, strengthening the hypothesis that early inflammatory changes impair the maternal metabolic adaptation required during pregnancy [9]. Retnakaran et al. further emphasized the mediating role of maternal adiposity, noting a strong correlation between CRP, adiposity indices, and insulin resistance, thus highlighting the need for comprehensive metabolic risk assessment [10].

In addition to CRP, other biochemical markers have also gained research attention. Elevated uric acid levels in early pregnancy have been associated with oxidative stress, endothelial dysfunction, and insulin resistance, factors central to GDM development. Likewise, first-trimester postprandial blood sugar (PPBS) levels, even within the normoglycemic range, may reflect subtle defects in glucose handling before fasting or oral glucose tolerance test (OGTT) abnormalities become apparent.

The Indian context provides a unique impetus for developing predictive models for early GDM detection. South Asian women exhibit a distinctive metabolic phenotype characterized by increased visceral adiposity, reduced insulin secretion, and heightened insulin resistance at comparatively lower BMI thresholds. This phenotype increases susceptibility to GDM and renders traditional screening strategies insufficient. Incorporating clinical risk factors - such as age, parity, family history of diabetes, and body mass index - with biochemical parameters like CRP, uric acid, and PPBS could significantly improve early prediction.

Therefore, there is a pressing need to move from reactive diagnosis during the second trimester toward proactive risk prediction in the first trimester. Developing integrated models that combine easily obtainable clinical variables with biochemical biomarkers offers an opportunity for timely intervention through lifestyle modifications, nutritional counseling, and pharmacological therapy when indicated. Such an approach could substantially improve maternal and fetal outcomes, while also reducing the long-term burden of type 2 diabetes and cardiovascular disease in Indian women and their children. The aim of the study was to evaluate the role of clinical and biochemical parameters during the first trimester as early indicators for the prediction of GDM.

## Materials and methods

Study design

The present study was designed as a prospective observational investigation, intended to monitor and analyze data as events unfolded naturally during pregnancy. This design was chosen because it allows researchers to observe temporal relationships between first-trimester biochemical markers and the subsequent development of GDM without intervening in the clinical management of participants. By prospectively following women from the first trimester through to the second trimester, the study sought to identify early predictors of GDM while minimizing recall bias and ensuring that data collection occurred in real time.

Study setting and duration

The study was conducted in the Department of Obstetrics and Gynecology at Sree Balaji Medical College and Hospital, Chromepet, Chennai, a tertiary care teaching institution located in South India. The hospital was selected as the study site because it caters to a large and diverse patient population, thus offering an appropriate setting for recruiting antenatal women across varying sociodemographic backgrounds. All infrastructure required for patient recruitment, clinical assessments, laboratory analyses, and ultrasonographic examinations was available within the institution, thereby facilitating consistent and reliable data collection. The study was carried out over a period of 18 months, ensuring adequate time for participant recruitment, baseline evaluations, and follow-up at 24-28 weeks of gestation.

Ethical approval and informed consent

Prior to initiation, the study protocol was reviewed and approved by the Institutional Ethics Committee of Sree Balaji Medical College and Hospital (002/SBMCH/IHEC/2023/2034). Ethical clearance was obtained in accordance with the principles outlined in the Declaration of Helsinki. All participants were enrolled only after providing written informed consent. The consent process included a detailed explanation of the study objectives, procedures, and potential risks or benefits. Participants were given adequate time to ask questions and clarify doubts before agreeing to participate. They were also assured of the confidentiality of their data and informed that they could withdraw from the study at any time without compromising the quality of their routine medical care.

Study population

The target population comprised antenatal women in their first trimester of pregnancy, defined as gestational age less than 14 weeks at the time of enrollment. A total of 118 participants were recruited consecutively during routine antenatal clinic visits, based on predetermined inclusion and exclusion criteria. Women were eligible if they had a confirmed gestational age of less than 14 weeks, were carrying a singleton pregnancy, and demonstrated fasting blood sugar levels below 92 mg/dL, ensuring that only women without overt hyperglycemia were enrolled. Participants also needed to express willingness to participate and provide informed consent.

Exclusion criteria were carefully defined to eliminate confounding factors that could interfere with glucose metabolism or biomarker levels. Women with known pregestational diabetes mellitus, multiple gestations, or chronic systemic illnesses such as renal disease, cardiovascular disease, connective tissue disorders, or chronic hypertension were excluded. Additional exclusions included women with gout, which could influence uric acid measurements, and those on medications known to induce hyperuricemia, such as pyrazinamide, ethambutol, levodopa, and theophylline. By applying these criteria, the study ensured a homogenous cohort, thereby strengthening the validity of the findings.

Sample size calculation

The sample size was calculated using the standard formula for estimating proportions. Based on a previous study, the expected prevalence of GDM in the first trimester was 7% [11]. With a 5% margin of error and 95% confidence level (1.96), the required sample size was calculated as 117.76, which was approximated to 118 participants and convenience sampling was done.

Clinical evaluation and data collection

At baseline, detailed sociodemographic and obstetric information was collected from each participant using a pretested data collection form. Clinical histories included maternal age, gravidity, parity, obstetric outcomes in prior pregnancies, and relevant family histories, particularly of diabetes mellitus and metabolic disorders. Histories of comorbid conditions, including polycystic ovary syndrome (PCOS), were also documented given their established association with metabolic dysfunction.

Anthropometric measurements were obtained at the time of enrollment. Height was measured to the nearest 0.1 cm using a stadiometer, and weight was recorded to the nearest 0.1 kg using a calibrated weighing scale. Body mass index (BMI) was calculated using the formula weight (kg)/height (m²). Blood pressure was measured using a mercury sphygmomanometer in the sitting position after a period of rest, and the average of two readings was recorded. These parameters provided baseline clinical data and served as potential risk factors for GDM.

Ultrasonographic confirmation of gestational age

To ensure accurate dating of pregnancy and proper classification of women into the first trimester, all participants underwent ultrasonographic examination. Ultrasonography also excluded cases with multiple gestations or uncertain viability. This step was critical in standardizing the cohort and eliminating confounding from gestational age misclassification.

Biochemical marker assessment

During the first trimester, venous blood samples were collected from all participants under strict aseptic precautions after an overnight fast of at least eight hours. The following biochemical parameters were measured: CRP: High-sensitivity CRP levels were quantified using an immunoturbidimetric assay and expressed in mg/L. This test was chosen for its sensitivity in detecting low-grade systemic inflammation. Serum uric acid: Levels were estimated using the uricase-peroxidase method and expressed in mg/dL. Uric acid served as a marker of oxidative stress and endothelial dysfunction. PPBS: This was measured enzymatically using the glucose oxidase-peroxidase method, approximately two hours after a standardized breakfast. All laboratory analyses were carried out in a National Accreditation Board for Laboratories (NABL)-accredited laboratory adhering to strict internal and external quality control protocols, ensuring reliability and reproducibility of results. The elevated thresholds are shown as CRP >8.5 mg/L; PPBS >130 mg/dL.

Follow-up and diagnosis of GDM

All participants continued to receive standard antenatal care throughout pregnancy. Between 24 and 28 weeks of gestation, each woman underwent a 75-gram oral glucose challenge test (OGCT) as per Diabetes in Pregnancy Study Group India (DIPSI) criteria. Plasma glucose was measured two hours after the glucose load, irrespective of fasting status. GDM was diagnosed if the two-hour plasma glucose concentration was ≥140 mg/dL. Based on these results, participants were stratified into GDM and non-GDM groups for further analysis.

Statistical analysis

Data entry was performed using Excel (Microsoft, Redmond, WA, USA), and statistical analyses were carried out using SPSS Statistics version 26.0 (IBM Corp., Armonk, NY, USA). Continuous variables, such as age, BMI, CRP, uric acid, and PPBS levels, were summarized as means with standard deviations, while categorical variables like parity, family history of diabetes, and history of PCOS were expressed as frequencies and percentages.

For group comparisons, the independent samples t-test was applied to continuous variables, while chi-square or Fisher’s exact tests were used for categorical variables as appropriate. To evaluate the predictive accuracy of biochemical markers in identifying women at risk of GDM, receiver operating characteristic (ROC) curve analyses were conducted. The area under the ROC curve (AUC) was calculated for CRP, uric acid, and PPBS individually. Optimal cut-off values were identified by maximizing sensitivity and specificity.

We pre-specified CRP, PPBS, BMI, PCOS, and family history of diabetes as candidate predictors based on prior literature. Continuous predictors were modelled as continuous; linearity in the logit was assessed using restricted cubic splines (4 knots at Harrell’s recommended quantiles). Multicollinearity was evaluated via variance inflation factors (VIF < 5). Discrimination was quantified by AUC with 95% confidence intervals estimated by DeLong’s method. Calibration was assessed using calibration plots and calibration slope/intercept; Hosmer-Lemeshow was reported as a supplementary statistic. Internal validation used bootstrap resampling (B = 1000) to obtain optimism-corrected estimates of AUC and calibration. Optimal thresholds were identified using Youden’s index; sensitivity, specificity, positive and negative predictive values were reported with 95% confidence intervals. Missing data were handled by multiple imputation by chained equations (m = 20), including all predictors and the outcome; estimates were pooled using Rubin’s rules. Given 31 events, we limited the effective degrees of freedom and performed sensitivity analyses with penalized logistic regression (ridge/LASSO). Two-sided p < 0.05 was considered significant.

## Results

The study population comprised 118 pregnant women, of whom 87 were non-GDM and 31 were diagnosed with GDM. The mean age of participants was comparable between the non-GDM (29.7 ± 3.0 years) and GDM groups (30.1 ± 3.3 years, p=0.18). Among non-GDM women, 27 (31.0%, 95%CI 9.2 -34.5) were primigravida and 60 (69.0%) were multigravida, while in the GDM group, nine (29.0%) were primigravida and 22 (71.0%) were multigravida (p=0.84). Anthropometric measures, including BMI (23.9 ± 4.5 vs. 24.2 ± 3.8, p=0.40), weight (61.4 ± 10.8 vs. 62.1 ± 9.5 kg, p=0.78), and height (159.8 ± 10.4 vs. 160.5 ± 9.3 cm, p=0.64), did not differ significantly between the groups. Major clinical risk factors were more prevalent among women with GDM, with 20 (64.5%) reporting a history of PCOS compared to 29 (33.3%) in the non-GDM group (χ²=9.20, p=0.004), and 18 (58.1%) having a family history of diabetes versus 25 (28.7%) among non-GDM women (χ²=18.25, p=0.003) (Table 1).

**Table 1:** Demographic, clinical characteristics, and risk factors among the study population (n=118) *Statistically significant at p < 0.05 Chi-square/ independent sample t test GDM: gestational diabetes mellitus, PCOS: polycystic ovary syndrome

Parameter	Non-GDM (n=87)	GDM (n=31)	t / χ² value	p-value
Age, years (Mean ± SD)	29.68 ± 2.97	30.12 ± 3.27	t = 1.36	0.18
Primigravida, n (%)	27 (31.0%)	9 (29.0%)	χ² = 0.04	0.84
Multigravida, n (%)	60 (69.0%)	22 (71.0%)		
BMI (Mean ± SD)	23.9 ± 4.51	24.2 ± 3.82	t = 0.85	0.40
Weight (Mean ± SD)	61.4 ± 10.8	62.1 ± 9.5	t = 0.28	0.78
Height (Mean ± SD)	159.8 ± 10.4	160.5 ± 9.3	t = 0.47	0.64
History of PCOS, n (%)	29 (33.3%)	20 (64.5%)	χ² = 9.20	0.004*
Family history of diabetes, n (%)	25 (28.7%)	18 (58.1%)	χ² = 18.25	0.003*

Biochemical parameters differed significantly between non-GDM and GDM women. PPBS was markedly higher in the GDM group (133.06 ± 13.15 mg/dL) compared to non-GDM women (92.6 ± 5.6 mg/dL, t = -14.0, p < 0.0001). Serum uric acid levels were also elevated in GDM participants (3.31 ± 0.52 mg/dL) versus non-GDM women (3.02 ± 0.28 mg/dL, t = -3.55, p = 0.02). Additionally, inflammatory marker CRP was significantly higher in the GDM group (12.96 ± 5.38 mg/L) compared to non-GDM women (5.51 ± 3.12 mg/L, t = -10.2, p < 0.0001). These results indicate that women with GDM had pronounced hyperglycemia, higher uric acid levels, and increased systemic inflammation relative to their non-GDM counterparts (Table 2).

**Table 2:** Association of biochemical parameters in non-GDM and GDM groups *Statistically significant at p < 0.05 Independent sample t test GDM: gestational diabetes mellitus, PPBS: postprandial blood sugar, CRP: C-reactive protein

Parameter	Non-GDM (Mean ± SD)	GDM (Mean ± SD)	t-value	p-value
PPBS (mg/dL)	92.56 ± 5.57	133.06 ± 13.15	t = -14.0	<0.001*
Uric acid (mg/dL)	3.02 ± 0.28	3.31 ± 0.52	t = -3.55	0.02*
CRP (mg/L)	5.51 ± 3.12	12.96 ± 5.38	t = -10.2	<0.001*

The diagnostic performance of biochemical markers for predicting GDM was evaluated using accuracy, AUC, precision, and F1 score. Among the markers assessed, CRP demonstrated the highest predictive performance with an accuracy of 0.91, an AUC of 0.94, precision of 0.92, and F1 score of 0.91. PPBS showed good diagnostic ability with an accuracy of 0.81, AUC of 0.85, precision of 0.83, and F1 score of 0.81. Uric acid also exhibited strong predictive potential with an accuracy of 0.79, AUC of 0.87, precision of 0.82, and F1 score of 0.81 (Table 3).

**Table 3:** Diagnostic accuracy of biochemical markers for predicting GDM GDM: gestational diabetes mellitus, PPBS: postprandial blood sugar, CRP: C-reactive protein, ROC: receiver operating characteristic, AUC: area under the ROC curve

Marker	Accuracy	AUC (ROC)	Precision	F1 Score
PPBS	0.81	0.85 (0.76–0.94)	0.83	0.81
Uric Acid	0.79	0.87 (0.79–0.95)	0.82	0.81
CRP	0.91	0.94 (0.88-0.98)	0.92	0.91

Table 4 shows that multiple regression analysis was conducted to evaluate the predictive value of biochemical markers for GDM. Uric acid alone showed minimal predictive ability (R² = 0.001, p = 0.876), while the combination of uric acid and CRP slightly improved prediction but did not reach statistical significance (R² = 0.042, p = 0.054). The model combining CRP and PPBS demonstrated strong predictive power, explaining 96.1% of the variance in GDM (R² = 0.691, p = 0.0024). The full model incorporating uric acid, CRP, and PPBS further increased predictive accuracy with R² = 0.790 and was highly significant (p < 0.0001). 

**Table 4:** Regression analysis of biochemical markers predicting GDM *Statistically significant at p < 0.05 GDM: gestational diabetes mellitus, PPBS: postprandial blood sugar, CRP: C-reactive protein

Model	R	R²	Std. Error	p-value
Uric acid	0.030	0.001	21.34	0.876
Uric acid + CRP	0.206	0.042	24.04	0.054
CRP + PPBS	0.980	0.691	20.00	0.0024*
Uric acid + CRP + PPBS	0.984	0.790	29.24	<0.0001*

Multivariate analysis revealed that several clinical and biochemical parameters were significantly associated with GDM. Women with a family history of diabetes had higher odds of developing GDM (OR 3.38, 95% CI 1.46-7.85, p = 0.004), as did those with a history of PCOS (OR 3.66, 95% CI 1.51-8.87, p = 0.003). Elevated CRP levels and PPBS were also strong predictors, with ORs of 6.21 (95% CI 2.01-19.18, p = 0.002) and 4.76 (95% CI 1.80-12.54, p = 0.002), respectively. Uric acid, however, was not a significant predictor in the multivariate model (p > 0.05) (Table 5).

**Table 5:** Multivariate analysis of demographic, biochemical and clinical parameters among the study groups *Statistically significant at p < 0.05 GDM: gestational diabetes mellitus, PCOS: polycystic ovary syndrome, CRP: C-reactive protein, PPBS: postprandial blood sugar

Clinical/Biochemical Parameter	Non-GDM n (%)	GDM n (%)	Odds Ratio (95% CI)	p-value
Family history of diabetes	25 (28.7%)	18 (58.1%)	3.38 (1.46–7.85)	0.004*
History of PCOS	29 (33.3%)	20 (64.5%)	3.66 (1.51–8.87)	0.003*
CRP (>8.5 mg/L)	5 (5.7%)	15 (48.4%)	6.21 (2.01–19.18)	0.002*
PPBS (>130 mg/dL)	7 (8.0%)	17 (54.8%)	4.76 (1.80–12.54)	0.002*
Uric acid (3.3 mg/dL)	8 (9.2%)	5 (16.1%)	1.23 (0.56 – 1.98)	0.45

The diagnostic performance analysis of three biochemical markers, CRP, PPBS, and uric acid, reveals that CRP (>8.5 mg/L) demonstrated the highest overall diagnostic accuracy, with a sensitivity of 83.9% (95% CI: 67.4-92.9%) and specificity of 87.4% (95% CI: 78.8-92.8%). Its high negative predictive value (93.8%, 95% CI: 86.4-97.3%) indicates that normal CRP levels reliably exclude disease presence, while a positive predictive value (70.3%, 95% CI: 54.2-82.5%) supports its role in confirming diagnosis when elevated. PPBS (>130 mg/dL) also performed well, showing a sensitivity of 80.6% (95% CI: 63.7-90.8%) and specificity of 85.1% (95% CI: 76.1-91.1%), with strong positive predictive value (65.8%) and negative predictive value (92.5%) values, suggesting its utility as a supplementary diagnostic marker. In contrast, uric acid (3.3 mg/dL) showed comparatively lower sensitivity (61.3%, 95% CI: 43.8-76.3%) and specificity (78.2%, 95% CI: 68.4-85.5%), indicating limited diagnostic reliability as a standalone marker, though its negative predictive value (85.0%, 95% CI: 75.6-91.2%) suggests it may still help in ruling out disease (Table 6).

**Table 6:** Diagnostic performance analysis of the biochemical markers in study CRP: C-reactive protein, PPBS: postprandial blood sugar, PPV: positive predictive value, NPV: negative predictive value

Marker (cut-off)	Sensitivity	95% CI	Specificity	95% CI	PPV	95% CI	NPV	95% CI
CRP (>8.5 mg/L)	83.9%	67.4–92.9%	87.4%	78.8–92.8%	70.3%	54.2–82.5%	93.8%	86.4–97.3%
PPBS (>130 mg/dL)	80.6%	63.7–90.8%	85.1%	76.1–91.1%	65.8%	49.9–78.8%	92.5%	84.6–96.5%
Uric acid (3.3 mg/dL)	61.3%	43.8–76.3%	78.2%	68.4–85.5%	50.0%	34.8–65.2%	85.0%	75.6–91.2%

## Discussion

The present study aimed to assess the predictive value of first-trimester biochemical and clinical markers - CRP, uric acid, PPBS, BMI, history of PCOS, and family history of diabetes - for GDM in a South Indian cohort. The incidence of GDM in our population was 27.9%, highlighting a substantial burden. Mean BMI was slightly higher among women who developed GDM compared to non-GDM controls (24.2 ± 3.82 vs. 23.9 ± 4.51 kg/m²), consistent with prior studies indicating that increased maternal BMI independently elevates GDM risk irrespective of age and parity [12]. Although the difference was not statistically significant, this trend underscores the importance of early BMI screening, particularly in South Asian women where metabolic risk manifests at lower BMI thresholds than in Western populations.

This study demonstrates that a composite assessment of first-trimester biochemical markers - CRP, uric acid, and PPBS - alongside key clinical risk factors such as PCOS and family history of diabetes, provides robust predictive accuracy for GDM. CRP and PPBS emerged as the strongest independent predictors, consistent with evidence linking low-grade inflammation and altered glucose metabolism to GDM pathogenesis [13]. While uric acid was significantly associated with GDM in univariate analysis, its predictive value diminished in multivariate models, suggesting that it is most informative within a comprehensive risk panel. The higher prevalence of PCOS and positive family history among GDM cases further emphasizes the importance of clinical risk stratification. The integration of these parameters yielded a highly predictive model (R² = 0.97), supporting early, multifactorial screening for GDM [14].

Inflammation has been implicated in GDM pathogenesis. CRP, a sensitive marker of systemic inflammation, was significantly higher in the GDM group (12.96 mg/L) than in non-GDM women (5.51 mg/L), suggesting that inflammatory processes may precede or accompany metabolic dysregulation in early pregnancy [13]. Traditional second-trimester GDM screening delays diagnosis, but early detection using biochemical markers can identify high-risk women before overt hyperglycemia develops [14]. In our cohort, first-trimester CRP and PPBS demonstrated strong predictive ability (AUC 0.94 and 0.85, respectively), indicating that incorporation of these markers into routine antenatal screening could facilitate timely interventions and improve pregnancy outcomes.

PCOS was present in 64.5% of GDM cases versus 33.3% of controls, consistent with other studies linking PCOS to insulin resistance and hyperandrogenism, both of which increase GDM risk [15,16]. Uric acid levels were higher in GDM women (3.31 ± 0.52 mg/dL vs. 3.02 ± 0.28 mg/dL, p=0.02), supporting its inclusion in early pregnancy risk panels, as suggested by similar studies, due to its associations with endothelial dysfunction, oxidative stress, and adverse pregnancy outcomes [17-19]. However, in multivariate analysis, uric acid did not retain independent significance, highlighting the greater predictive power of combined clinical and biochemical models.

CRP emerged as the most robust single biochemical predictor, echoing prior work by Kumari R et al. and Buchanan and Xiang, which implicates low-grade inflammation in the metabolic adaptations that fail in women who develop GDM [20,21]. Family history of diabetes also demonstrated a strong association, present in 58.1% of GDM cases versus 28.7% of controls, reinforcing the role of heritable metabolic traits in GDM pathogenesis and supporting its routine inclusion in early screening protocols [22]. PPBS in the first trimester was markedly higher in GDM women (133.06 ± 13.15 mg/dL vs. 92.56 ± 5.57 mg/dL, p<0.0001), consistent with evidence that even mild gestational hyperglycemia negatively impacts pregnancy outcomes [23]. Beyond generic machine learning (ML), a 2025 biomarker study and a parallel medicine analysis both show that broader cardiometabolic panels can improve discrimination while raising issues of assay availability, cost, and clinical integration - highlighting the trade-off between simplicity (our CRP+PPBS) vs. complexity (multi-analyte panels/ML) [23,24].

The combination of clinical and biochemical markers yielded the highest predictive accuracy, emphasizing the need for multifactorial screening approaches. Such models are particularly relevant in resource-constrained settings like India, where targeted early intervention can yield substantial benefits [25]. Early identification allows for lifestyle modification, dietary counseling, and, when necessary, pharmacological management, improving maternal and neonatal outcomes, as demonstrated by Landon et al. [26]. Metabolic and inflammatory changes detectable in the first trimester have downstream effects on maternal and fetal health, underscoring the importance of early risk assessment [27].

This study has a few limitations. The single-center design and modest sample size may limit generalizability, particularly outside South India. Self-reported histories for PCOS and family diabetes risk introduce potential recall bias. Residual confounding from unmeasured variables, including diet, physical activity, and socioeconomic status, cannot be excluded. The study did not assess other emerging predictors such as adipokines, genetic markers, or iron status, which may influence both inflammation and glucose metabolism. Finally, long-term maternal and neonatal outcomes were not tracked, precluding assessment of the full clinical impact of early GDM prediction.

Despite these limitations, our findings are hypothesis-generating and demonstrate associations between first-trimester CRP/PPBS and DIPSI-defined GDM within a single tertiary-care cohort. Given the small number of GDM events (n=31), potential overfitting of multivariable models, and the use of DIPSI criteria (which may classify GDM differently from IADPSG/ADA), these results should not be interpreted as sufficient evidence for clinical implementation. The marker panel requires external, multicentre validation, assessment of calibration and transportability under alternative diagnostic standards, and decision-curve/impact and cost-effectiveness analyses before any consideration for routine screening. Future multicentric studies with larger cohorts and additional biomarkers are warranted to validate and refine predictive models for broader clinical application.

## Conclusions

First-trimester assessment of biochemical markers, particularly CRP and PPBS, combined with clinical risk factors such as history of PCOS and family history of diabetes, provides a robust and early prediction model for GDM in South Indian women. While uric acid showed limited independent predictive value, it contributes to risk stratification when included in a comprehensive panel. Early identification of high-risk women enables timely interventions, including lifestyle modification and targeted monitoring, potentially reducing adverse maternal and neonatal outcomes. Integrating these markers into routine antenatal screening may improve GDM detection and management, especially in populations with high baseline metabolic risk. Accordingly, these findings are hypothesis-generating and do not justify clinical implementation at this stage. External, multicentre validation-ideally across community and tertiary settings and under alternative diagnostic standards (e.g., IADPSG/ADA)-with full reporting of calibration, transportability, and clinical utility (decision-curve and cost-effectiveness analyses) is required before any consideration of practice adoption.
